# Systematic clinical approach for diagnosing upper limb tremor

**DOI:** 10.1136/jnnp-2019-322676

**Published:** 2020-05-26

**Authors:** Jaron van de Wardt, A M Madelein van der Stouwe, Michiel Dirkx, Jan Willem J Elting, Bart Post, Marina AJ Tijssen, Rick C Helmich

**Affiliations:** 1 Department of Neurology, Canisius Wilhelmina Ziekenhuis, Nijmegen, The Netherlands; 2 Department of Neurology, University Medical Centre Groningen (UMCG), Groningen, The Netherlands; 3 Expertise Center Movement Disorders Groningen, University Medical Center Groningen (UMCG), Groningen, The Netherlands; 4 Department of Neurology, Donders Institute for Brain, Cognition and Behaviour, Radboud University Nijmegen Medical Center, Nijmegen, The Netherlands; 5 Department of Clinical Neurophysiology, University Medical Center Groningen (UMCG), Groningen, The Netherlands

**Keywords:** tremor, parkinson's disease, dystonia, myoclonus, neurophysiol, clinical

## Abstract

Tremor is the most common movement disorder worldwide, but diagnosis is challenging. In 2018, the task force on tremor of the International Parkinson and Movement Disorder Society published a consensus statement that proposes a tremor classification along two independent axes: a clinical tremor syndrome and its underlying aetiology. In line with this statement, we here propose a stepwise diagnostic approach that leads to the correct clinical and aetiological classification of upper limb tremor. We also describe the typical clinical signs of each clinical tremor syndrome. A key feature of our algorithm is the distinction between isolated and combined tremor syndromes, in which tremor is accompanied by bradykinesia, cerebellar signs, dystonia, peripheral neuropathy or brainstem signs. This distinction subsequently informs the selection of appropriate diagnostic tests, such as neurophysiology, laboratory testing, structural and dopaminergic imaging and genetic testing. We highlight treatable metabolic causes of tremor, as well as drugs and toxins that can provoke tremor. The stepwise approach facilitates appropriate diagnostic testing and avoids unnecessary investigations. We expect that the approach offered in this article will reduce diagnostic uncertainty and increase the diagnostic yield in patients with tremor.

## Introduction

**Figure 1:**
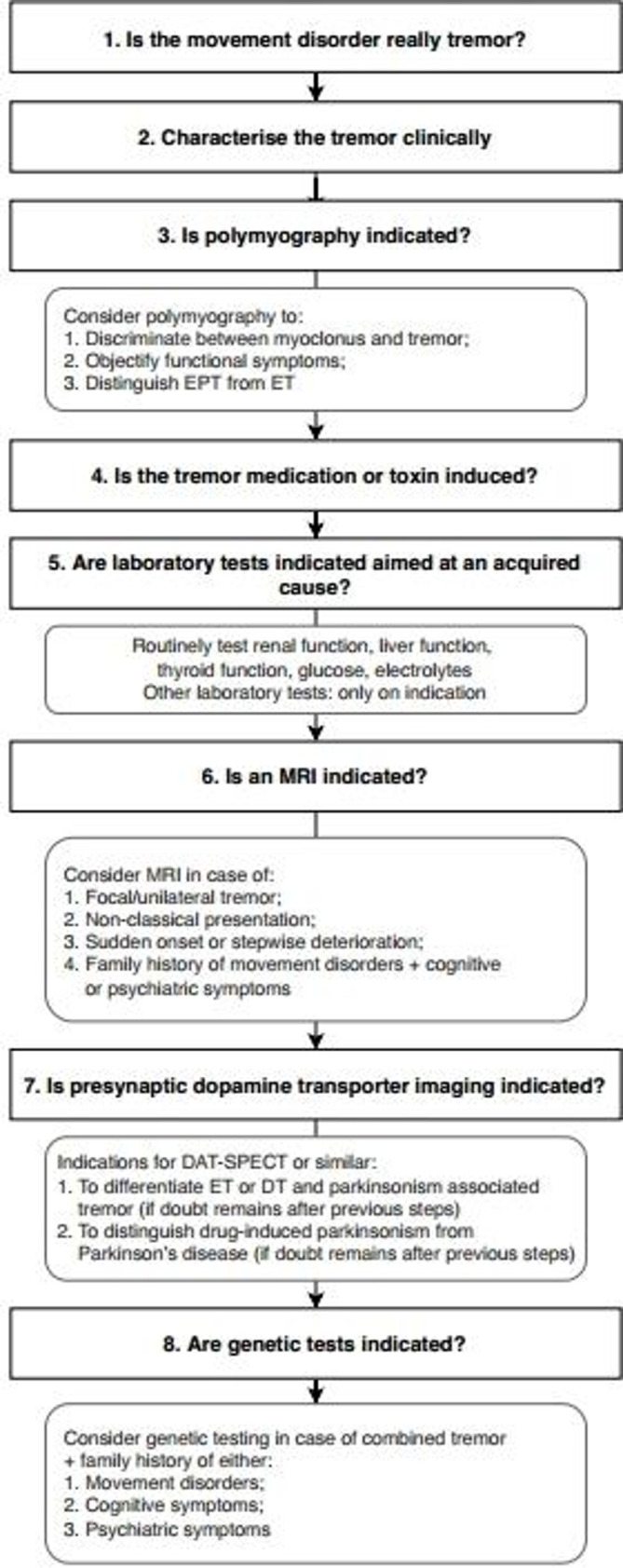
Eight-step diagnostic approach to upper limb tremor. DT, dystonic tremor; EPT, enhanced physiological tremor; ET, essential tremor.

Although tremor is the most common movement disorder and therefore frequently encountered by neurologists,^1^ diagnosing tremor accurately can be challenging. The clinical presentation of a patient with tremor can range from a relatively isolated symptom to a complex syndrome, incorporating multiple signs and symptoms. In 2018, the task force on tremor of the International Parkinson and Movement Disorder Society published a consensus statement in which a tremor classification along two independent axes is proposed^2^: determination of a clinical syndrome based on clinical features (axis 1), and determination of an aetiology (axis 2). For example, a bilateral upper limb action tremor without other neurological signs is classified as essential tremor (ET) on axis 1. The same patient may have a mutation in a gene that often causes dystonia; this is classified as the aetiology on axis 2. However, the axis 1 diagnosis of ET is not changed until dystonia develops (if it ever does).^3^ In the current paper, we propose a practical approach in line with the consensus statement, to aid practising clinicians in diagnosing patients with tremor. While this approach has not been tested yet, it is based on the current literature and in line with the steps taken at our Expertise Centres for Movement Disorders. The first three steps we propose will lead to identification of a clinical syndrome, corresponding to axis 1. The subsequent steps will help in establishing an aetiology where possible (axis 2). Because of its prevalence and diagnostic complexity, we will focus on visible upper limb tremor, and omit discussion of related movement disorders such as palatal tremor and orthostatic tremor. Our aims are to provide organising principles on how to interpret clinical symptoms, and to provide guidelines for diagnostic testing, in which we recommend what is indicated while steering clear from unnecessary investigations. We acknowledge that many of these recommendations are based on expert opinion. This reflects the current state of the field, given that many clinical tremor syndromes lack a gold standard against which clinical signs or ancillary investigations can be tested. A systematic approach to phenotype patients with upper limb tremor will, however, make it easier to test and validate new biomarkers in future research. Our stepwise approach to tremor diagnosis is presented in figure 1.

### Step 1: is the movement disorder really tremor?

The first step in diagnosing tremor is the identification of hyperkinetic movements as ‘tremulous’. Tremor is defined as an involuntary, rhythmic, oscillatory movement of a body part.^2^ The most important alternative diagnosis is myoclonus, especially high frequency cortical myoclonus (e in familial cortical myoclonus), polyminimyoclonus (eg, in multiple system atrophy), peripheral myoclonus (which sometimes occurs in inflammatory neuropathies or radiculopathies) or asterixis (a negative myoclonus that looks like a flapping tremor of the hands when the wrists are extended).^4 5^ Moreover, the rhythmic, low-frequency jerks in ongoing focal epilepsy (epilepsia partialis continua) can be misinterpreted as unilateral jerky rest tremor.^4^ Generally, a ‘jerky’ aspect ought to raise suspicion of myoclonus as the movement disorder in question, rather than tremor. However, the distinction between high frequency myoclonus and tremor on the basis of ‘rhythmicity’ is complicated by the fact that tremor is almost never *perfectly* rhythmic. In fact, a jerky or irregular aspect is part of the characteristic phenotype of some tremor syndromes, such as dystonic tremor or Holmes tremor.^6^ When in doubt, polymyography can be used to differentiate between myoclonus and tremor: polymyography is further described in step 3. Naturally, tremor and myoclonus are not mutually exclusive: patients can have both movement disorders.

### Step 2: characterise the tremor clinically

**Figure 2:**
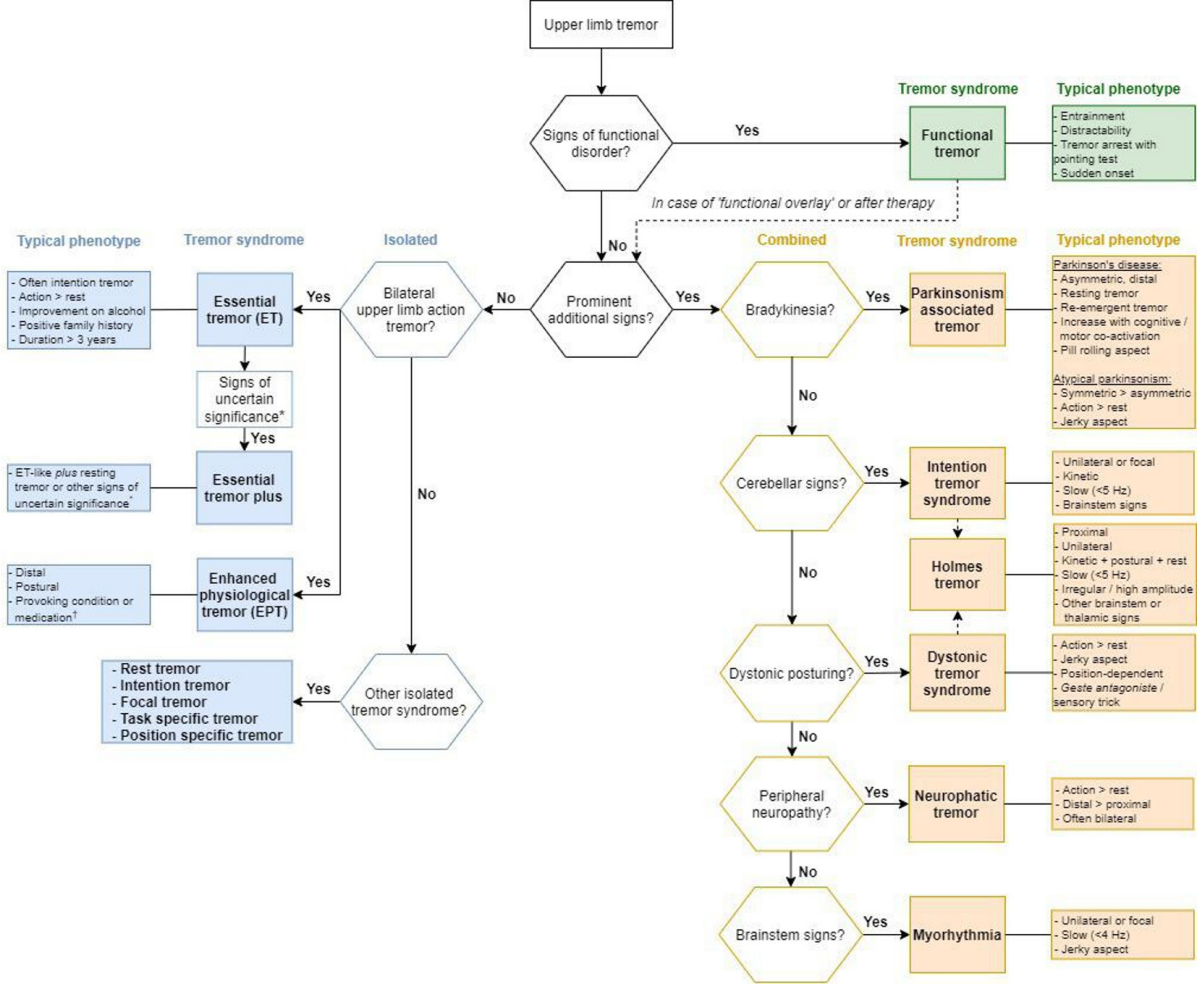
Practical clinical algorithm. Isolated tremor syndromes are depicted in blue on the left side of the diagram, combined tremor syndromes in orange on the right side. Functional tremor is depicted in green on the right side. *For example, mildly unsteady tandem gait, questionably abnormal posturing of a body part, mild cognitive impairment, questionable bradykinesia or rigidity and other mild neurological signs of unknown significance that do not suffice to make an additional syndrome classification or diagnosis. †For example, anxiety, fatigue, thyrotoxicosis, hypoglycaemia, pheochromocytoma, tremor-provoking medication (see table 1).

The neurological examination is the key tool to establish a clinical tremor syndrome, that is, an axis 1 classification. This is of crucial importance, as the tremor syndrome is used in later steps to guide our choices for diagnostic tests to elucidate the underlying aetiology (ie, an axis 2 classification). In figure 2, we propose a practical clinical algorithm that helps to accurately diagnose a tremor syndrome. Before entering this algorithm, it is useful to characterise the tremor using the features outlined in box 1. The algorithm contains decision points in an order that facilitates ‘short-cuts’ to the diagnosis of a clinical tremor syndrome.

**Table 1:** Overview of medication and toxins that may cause tremor^52 53^

Medication	Toxins
Sympathomimetic drugs (adrenaline epinephrine, bronchodilators, theophylline)	Drugs of abuse (nicotine, alcohol and its withdrawal, cocaine, caffeine)
Antiarrhythmics (amiodarone)	Lead
Metoclopramide	Mercury
Antidepressants (SSRI, TCA)	Manganese
Lithium	Arsenic
Neuroleptics, tetrabenazine	Cyanide, DDT, CO
Antiepileptic drugs (valproic acid, carbamazepine, phenytoin)	Naphthalene
Immunosuppressants (tacrolimus, ciclosporin)	Toluene
Anticancer drugs (vincristine, cisplatin, paclitaxel, doxorubicin, cytosine arabinoside, ifosfamid, tacrolimus, 5-fluorouracil, methotrexate, tamoxifen, cytarabine)	Lindane
Hormones (thyroxine)	Solvents
Steroids (progesterone, anti-estrogens, adrenocorticosteroids)	Pesticides

CO, carbon monoxide; DDT, dichlorodiphenyltrichloroethane; SSRI, selective serotonin reuptake inhibitor; TCA, tetracyclic antidepressants.

Box 1 **Examination of a patient with tremor**

**General guidelines:**

- View straight from in front, such that both arms can be seen simultaneously.
- Assess tremor for >5 s per condition, ideally 30 s per position.

**Activation conditions:**

- *Rest*. This is ideally done when the subject lies completely relaxed on a bed, especially for (rest) tremor of the head and tremor of the lower limbs. If not possible, use an armchair (hands dangling down from the armrests, forearm supported) or ask the patient to have both forearms pronated in a relaxed position.
- *Rest with cognitive co-activation*. Ask the patient to perform serial sevens, name as many animals as possible or spell a difficult word. Note increased tremor amplitude (eg, in Parkinson’s tremor) or reduced tremor amplitude (eg, in functional tremor).
- *Rest with motor co-activation*. Ask the patient to make tapping movements with the contralateral hand. Look for resting tremor in the arms during walking. Note increased tremor amplitude (eg, in Parkinson’s tremor) or reduced tremor amplitude (eg, in functional tremor).
- *Change from rest to posture (to identify re-emergent tremor)*. Ask the patient to rapidly extend the wrist and note whether this leads to tremor reset (a transient (>1–2 s) suppression of the tremor amplitude). This is often seen in Parkinson’s tremor.
- *Postural tremor*. Look for tremor when the arms are extended towards you (palms down), when the hands are supinated and when the hands are pronated. Note position dependence (eg, in dystonic tremor). Ask the patient to spread the fingers, and note myoclonic movements of individual fingers (eg, in polyminimyoclonus). Look for tremor during the wing-beating position (both hands before the mouth, not touching each other). Note involvement of proximal shoulder muscles in the tremor during this position (eg, in dystonic tremor or Holmes tremor). Ask and test for position-dependent tremors, for example, when a cupis held in a particular position. Test for stimulus-sensitivity of the hyperkinetic movement by briefly tapping the fingers at the palmar side during posturing (if the movements increase, this may indicate cortical myoclonus).
- *Kinetic tremor*. Ask the patient to make slow finger-to-nose movements. Distinguish between simple kinetic tremor (tremor amplitude remains constant during the entire movement) and intention tremor (crescendo increase in tremor when affected body part approaches its visual target). Ask and test for task-specific kinetic tremor (eg, during writing). Ask patients to draw a spiral with each hand separately, unsupported by the table.
- *Note tremor in other locations than the arms*. For example, the head, face, tongue, voice, legs, trunk.

**Signs of functional tremor**

- *Note abrupt changes in tremor movement*. For example, changing from wrist flexion/extension to pronation/supination, from left to right or between body parts.
- *Note distractibility*. Look for disappearance of tremor during cognitive or motor co-activation. Also note inability of the patient to perform simple tasks.
- *Note entrainment*. Ask the patient to make tapping movements with the contralateral hand at different frequencies, and note whether the tremor rhythm adopts these frequencies. Distinguish entrainment from mirror movements (which occur in many movement disorders).
- *Perform pointing test*. Ask the patient to keep the tremulous limb in the position where the tremor occurs (eg, stretched out), move it slightly out of view of the patient and focus on the contralateral arm. Ask the patient to make a rapid, ballistic movement with that arm (eg, grab the examiner’s finger). The pointing test if positive if the tremor is briefly suppressed during the movement of the contralateral arm.

First, it is useful to look for signs of a functional movement disorder. Tremor is a common phenotype of functional movement disorders.^7^ Signs to investigate are entrainment (evaluate whether the tremor frequency changes when the patient performs a contralateral finger tapping task), distractibility (cessation of tremor on distraction by a mental or contralateral movement task) and temporary arrest of tremor during a ballistic pointing test of the other hand (‘pointing test’). Inability to perform a simple cognitive task can also be a sign of a functional movement disorder, as well as a sudden onset of the tremor.^8^ Sometimes marked fluctuations of the frequency of a functional tremor (between different contexts or spontaneously) can be noticed by visual inspection alone, otherwise polymyography can be used (step 3). Finally, the examiner may influence the co-contraction pattern by moving the patient’s hand passively and note whether or not the tremor fluctuates in parallel to the muscle tone.^9^ This can be done during clinical assessment or during polymyography (allowing a quantification of muscle contraction and tremor amplitude). An important pitfall is that functional movement disorders may occur in patients who also have an organic movement disorder.

Second, we suggest looking for so-called ‘prominent additional signs’. This distinction is important, because tremor can occur in isolation (*isolated* tremor syndromes) or together with other neurological signs (*combined* tremor syndromes). A distinction is made between ‘prominent additional signs’ and so-called ‘signs of uncertain significance’.^2^ The first are clear neurological signs, used to define a combined tremor syndrome, such as bradykinesia, cerebellar signs, dystonic posturing, signs of peripheral neuropathy and brainstem signs. Examples of ‘signs of uncertain significance’ include a mildly unsteady tandem gait, questionably abnormal posturing of a body part, mild cognitive impairment, questionable bradykinesia or rigidity and other mild neurological signs that do not suffice to make an additional syndrome classification.^2^ As can be seen in the algorithm, the weight placed by the clinician on an additional symptom (such as dystonic posturing of a limb) determines whether a tremor is called dystonic tremor or ET ‘plus’. This can be difficult, as outlined under ‘Isolated tremor syndromes’ section.

#### ​Combined tremor syndromes

In the right column of the algorithm, the *combined* tremor syndromes are depicted. In classifying a combined tremor syndrome, there are five prominent additional signs to look for.

Bradykinesia points to a parkinsonism-associated tremor syndrome, such as Parkinson’s disease (PD) or atypical parkinsonism (progressive supranuclear palsy (PSP), multiple system atrophy (MSA), vascular parkinsonism, dementia with Lewy bodies, corticobasal degeneration or medication-induced parkinsonism). Tremor in PD (Parkinson’s tremor) is often distal, asymmetric, it occurs predominantly at rest and has a frequency of 4–7 Hz. The tremor can show a ‘pill-rolling’ aspect (eg, more flexion than extension of the thumb), often spontaneously waxes and wanes in amplitude and can improve on levodopa—although not necessarily.^10^ Parkinson’s tremor typically increases in amplitude during cognitive co-activation (eg, serial sevens) or motor co-activation with another limb (eg, tapping with the contralateral hand, or walking).^11^ It classically occurs at rest, but a lot of patients also have postural tremor (46%–93%). In 81% of these patients, a transient (>1–2 s) suppression of tremor amplitude is seen during a voluntary movement with the trembling arm, after which the tremor comes back (re-emergent tremor).^12^ Although resting tremor is a key feature of a Parkinson’s tremor, 19% of the patients show an additional pure postural tremor that is independent from the resting tremor (unlike re-emergent tremor, which is a continuation of resting tremor during stable posturing).^10^ Tremor occurs less often in atypical parkinsonism than in PD, but it can nevertheless be a presenting sign. In comparison, tremor in MSA is often jerkier and lacks the classical ‘pill-rolling’ aspect of rest tremor in PD (12% in MSA vs 74% in PD), while postural, action and intention tremor are more frequently seen in MSA.^13^ Tremor in PSP is also more often present during actions than during rest and it is usually symmetrical as opposed to asymmetrical tremor PD, although a PD-like tremor has also been reported.^14^

Cerebellar signs are seen in intention tremor syndromes. The main cerebellar sign is ataxia, which can become visible as dysmetria of the limbs or eye movements, dysarthria, dysdiadochokinesia and unsteady broad-based gait. Clinically, the tremor syndrome consists of a unilateral or focal, kinetic and slow (<5 Hz) (intention) tremor, which can be accompanied by brainstem signs (eg, diplopia, cranial nerve palsy). If there are no cerebellar or brainstem signs, but only an intention tremor, the diagnosis of isolated intention tremor can be made, though this is very rare.^2^

Dystonic posturing is the key feature of a dystonic tremor syndrome. It is now recognised that tremor is a very common feature of dystonia, occurring in 17%–55% of patients with adult-onset primary dystonia.^15 16^ A distinction can be made between ‘dystonic tremor’, in which dystonia in the affected limb is found (eg, head tremor in a patient with cervical dystonia), or ‘tremor associated with dystonia’, in which dystonia is found in any other body part than the tremulous limb (eg, hand tremor in a patient with cervical dystonia).^2^ Dystonic tremors are mostly postural or kinetic, but they may also occur at rest. The tremor usually has a jerky or irregular aspect, and it can exacerbate by attempting to maintain a certain posture.^16 17^ Sometimes a *geste antagoniste* (sensory trick) can be found, which helps in differentiating dystonic head tremor from essential head tremor,^18^ as does a rest tremor of the head, which seems more common in dystonic head tremor. Usually, no improvement on levodopa is seen.

Peripheral neuropathy, consisting of distal lower motor neuron signs (muscle weakness and atrophy, absent reflexes) and distal sensory signs, suggests a neuropathic tremor. This tremor is mainly postural, but can also be found at rest, and is most frequently found in inflammatory neuropathies like IgM paraproteinemic neuropathies, chronic inflammatory demyelinating neuropathy and multifocal motor neuropathy.^19^ In very rare cases, tremor is associated with a peripheral radiculopathy^20^ or with certain myopathies.^21^

Brainstem signs are relevant as they can be found in patients with myorhythmia. This is a very rare movement disorder characterised by rhythmic movements of the cranial or limb muscles are seen in rest or during action. It is unilateral or focal, of slow frequency (<4 Hz) and often has a jerky aspect. Because of its irregular clinical features, it does not conform to the traditional definitions of tremor. Therefore, some have argued to consider myorhythmia as a separate entity.^22^ Oculo-masticatory myorhythmia is nearly pathognomonic in patients with Whipple disease, and can be accompanied by a slow convergent-divergent nystagmus and a vertical ophthalmoparesis.^23^

Finally, patients may present with multiple additional signs. For example, 30%–60% of patients with PD also have dystonic posturing.^24^ In Holmes tremor, many patients have additional signs of ataxia and/or dystonia. Holmes tremor is a slow (<5 Hz), unilateral, more proximal tremor, which is seen during actions and at rest. The tremor usually has a high amplitude, is irregular in appearance and often involves proximal muscles. Furthermore, it can be accompanied by brainstem or thalamic signs (eg, lateralised sensory disturbances), because a lesion is usually found in these locations.^25^

#### ​Isolated tremor syndromes

In the left column of the algorithm, the *isolated* tremor syndromes are depicted.

The most likely diagnosis in a patient presenting with a bilateral upper limb action tremor is ET, as it has the highest prevalence, and the prevalence and severity increases with age. Bilateral action tremor is mandatory for this diagnosis. Formally, this tremor syndrome has at least 3 years of duration, (otherwise it ought to be called an isolated action tremor), without a sudden onset, stepwise deterioration or task or position specificity. The tremor can spread to other locations (eg, head, voice or lower limbs). It improves with the use of alcohol in 50% of the cases, although it should be noted that alcohol can also reduce other tremors due to its sedative effects. Furthermore, self-reported alcohol responsiveness correlates poorly with actual objectified responsiveness,^26^ so a history of alcohol sensitivity has limited value as a diagnostic tool. Half of the ET patients report a positive family history.^27^ Of the patients diagnosed with an ET syndrome at a tertiary referral centre, 42% had an intention tremor.^28^ In severe cases, a rest tremor can also be found (around 18%).^29^ In advanced disease, tandem gait abnormalities can be found.^30^

If there are additional signs ‘of uncertain significance’ in a patient who otherwise fulfils the criteria for ET, then the recent consensus classifies this as ET plus. This could be impaired tandem gait, questionable dystonic posturing, memory impairment or other mild neurological signs. Likewise, ET with tremor at rest should be classified here.^2^ Over the last few years, there has been considerable discussion over the term ‘ET plus’. A recent study showed that, in ET patients with lower limb tremor, 110/133 (83%) were re-classified as ET plus, and these patients were older than the ET group.^31^ This shows that many patients with ET have additional symptoms, which may be integral to the disease, reflect comorbidity or it may be related to ageing. Another issue is that it is not easy to determine the clinical significance of additional symptoms. Specifically, two recent studies have shown that there is only fair agreement between movement disorders experts when assessing soft signs,^32^ and modest inter-rater agreement between axis 1 diagnoses (especially between ET plus and dystonic tremor).^33^ Neurophysiological measures, such as temporal discrimination thresholds, also cannot provide clear distinctions between ET and dystonic tremor, although this may be useful when classifying indeterminate tremors.^34^ These findings show that the borders between some clinical tremor syndromes are not fixed, which may indicate a shared pathophysiological basis.^3^ Accordingly, it has been argued that ‘ET plus’ may represent a state condition, such that additional signs emerge during certain stages of ET,^35^ or a temporary label, such that patients with ET plus transition to a more definite diagnosis.^36^ In the future, incorporation of pathophysiological information (a potential ‘third axis’) may help to better classify tremor syndromes, but this requires better biomarkers than are currently available. Until then, we recommend to not rest at a diagnosis of ‘ET plus’, and to explicitly note and follow the relevant ‘plus symptom’. The general principle that axis 1 classifications may change over time is, however, not specific to ET plus.

A second consideration is whether the clinical phenotype fits the diagnosis of enhanced physiological tremor (EPT). The limbs and head, when unsupported, exhibit slight tremor in every living person,^37^ and it is this physiological tremor that is exacerbated in EPT. When taking the patient’s history, search for an influence of provoking factors such as anxiety, fatigue, thyrotoxicosis, hypoglycaemia, pheochromocytoma or tremor inducing medication. The typical phenotype consists of a bilateral, distal, mostly postural tremor, which is a little faster than ET, and has a lower amplitude than ET.^38^ The diagnosis is confirmed by normalisation of the tremor after management of the provoking condition.^2^

The last group of isolated tremors consists of other isolated tremor syndromes. These syndromes might develop over time into a *combined* tremor syndrome, for example, primary writing tremor may evolve to dystonic tremor syndrome, or an isolated rest tremor may be precursor of PD. We distinguish four other isolated tremor syndromes^2^:

1. Isolated rest tremor: rest tremor without other clinical features (such as bradykinesia or dystonia) most commonly occurs in an upper or lower limb or as a hemitremor, but it may occur elsewhere (eg, lips, jaw or tongue). If an isolated rest tremor has the clinical features of a Parkinson tremor (figure 2), nuclear imaging may be considered if there is doubt about the presence or absence of bradykinesia (step 7).
2. Isolated intention tremor: intention tremor <5 Hz without localising signs, which is very rare.
3. Isolated focal tremors (voice, head, jaw, face, others), for example, isolated voice tremor: visible and/or audible tremor of the vocal apparatus (not in the context of dystonia) or isolated head tremor: shaking of the head like yes-yes or no-no, or variable directions, etc.
4. Isolated task-specific and position-specific tremors (writing, sports, musicians): occur during a specific task or posture (eg, primary writing tremor or *yips* in golfers).

### Step 3: is polymyography indicated?

Polymyography can be used to support the suspected clinical diagnosis or advance the clinical characterisation of the tremor. However, the value depends on the skills and experience of the neurophysiologist. Therefore, the availability of polymyography varies between centres and the quality is highest in specialised clinics. This can be a reason for referral of particularly difficult cases. There are a number of specific situations in which it can be of help.

First, polymyography can be used to make the distinction between tremor and myoclonus. Look for characteristics of (cortical) myoclonus such as 1) short electromyogram (EMG) bursts (<50–100 ms), 2) at an irregular frequency, 3) in a synchronous agonist-antagonist contraction pattern, 4) or transient loss of muscle tone (negative myoclonus). More advanced techniques, such as back-averaging and coherence analysis, include a combination of EMG and electroencephalogram (EEG) in search for signs of cortical drive.^39–41^ It should be borne in mind that epilepsia partialis continua is not always associated with epileptic EEG activity: in ±17% of cases, the ictal EEG is normal.^42^

A second useful application is the objective assessment of signs associated with functional tremor, such as entrainment and distractibility (step 2). Functional tremor can present as an alternating tremor or as a co-contraction tremor. A co-contraction tremor (where agonist and antagonist muscles increase limb stiffness, resulting in a fine, high-frequency tremor) can be more resistant to entrainment and distraction. In these cases, polymyography can be used to identify the co-contraction pattern, which is typically present at the onset of posturing. Furthermore, the pointing test is often positive in co-contraction tremor, and polymyography is more sensitive to detect this than the clinical eye. In 2016, a test battery was validated as laboratory-supported criteria for the diagnosis of functional tremor, including an increase of tremor amplitude on loading, tonic discharge of antagonist muscles approximately 300 ms before tremor onset, entrainment or increase in variability and change of tremor frequency while tapping with the contralateral hand, less accurate tapping performance at requested frequencies, significant interlimb coherence in bilateral tremors and transient arrest of tremor during ballistic movements of the other hand.^43^ By application of this test battery at a cut-off score of ≥3/10 points, functional tremor can be distinguished from organic tremor with a sensitivity of 90% and a specificity of 96%. While many of these tests can be done clinically, polymyography is usually more sensitive and in some cases necessary, for example, for calculating coherence. Moreover, spontaneous and marked (>1 Hz) variability in tremor frequency throughout the registration is also a marker of functional tremor.^28 44^ It is important to appreciate that while most tests for functional tremor have a high specificity, none are 100% specific. In other words, ‘functional’ characteristics can occasionally occur in otherwise ‘organic’ tremor, and it is of course possible that an existing organic tremor is aggravated by functional tremor. Complimentary to the establishment of functional characteristics, polymyography can be an important identifier of tremor with an unusual clinical appearance. These unusual tremors are sometimes incorrectly recognised as functional tremor, as can happen in lesion-based tremors such as Holmes tremor or myorhythmia.^45^

A third use of polymyography is to differentiate between EPT and ET. EPT has mechanical and mechanical-reflex components but only rarely central components (±10%), while ET has a central origin. Mechanical components are body part oscillations that are driven by factors such as cardiac systole, breathing and irregularities in muscle contraction. These mechanical components may trigger a reflex response in the muscle under certain circumstances (eg, fatigue, stress, intake of adrenergic medication). The resulting tremor can be influenced by mechanical factors such as inertial loading or by changing the movement tasks or postures. This introduces changes in stiffness or the contribution of different joints, which influences the tremor frequency. If a change in these factors leads to a change in tremor frequency, this points towards EPT.^46^ A decrease in tremor frequency after inertial loading is a specific finding^47 48^ (see Longardner *et al* for an instructional video on how to conduct this test properly).^49^ A higher frequency (>8 Hz) also points towards EPT.

Finally, polymyography may be helpful when selecting appropriate muscles for botulinum toxin treatment in patients with (dystonic) tremor. Polymyography can also confirm the low frequency (<5 Hz) that is typical of Holmes tremor, as frequency is difficult to judge by eye.^50 51^

### Step 4: is the tremor medication or toxin induced?

After clinical phenotyping as described in steps 1–3, the next steps provide a systematic way to search for an aetiology. Use of tremorgenic medication is a common cause of mainly enhanced physiological and parkinsonism-associated tremor: the tremor emerges after the start or dose escalation of the offending drug and vanishes usually shortly after discontinuation.^52^ An overview of medicines and toxins that are known to cause tremor is provided in table 1.^53^ A tremor produced by lithium, antidepressants or toxins frequently has the clinical appearance of rhythmic cortical myoclonus. Exposure to toxins is common in certain occupations and tremor can be the first manifestation in a constellation of exposure toxicity signs and symptoms. It is important to recognise these acquired causes of tremor, because reduction or removal of the medication or toxic agent is the first step in treating these patients.

### Step 5: are laboratory tests indicated aimed at an acquired cause or treatable metabolic disorder?

Tremor and myoclonus can be caused by forms of organ failure or homeostatic imbalance, such as renal failure, hepatic failure, hyperthyroidism and disturbed glucose metabolism. These causes are important to identify, as treatment leads to cessation of tremor. Basic laboratory testing is therefore advised, especially in patients with a bilateral isolated tremor syndrome, and this should include thyroid, liver and renal function, glucose and electrolytes. The exact yield of these tests is, however, unknown. Other tests should only be performed when they are specifically indicated, to minimise unsolicited findings. In *isolated* tremor, investigate serum catecholamines if there are other symptoms of pheochromocytoma (ie, episodes of headache, sweating and/or tachycardia).^54^ Test for toxins when there is a history of exposure.^53^ In tremor *combined* with dystonia or a family history of movement disorders and cognitive or psychiatric symptoms, consider testing copper and iron metabolism (serum copper, ceruloplasmin, iron, ferritin) as this can point to Wilson disease^55^ or neurodegeneration with brain iron accumulation^56^ (particularly neuroferritinopathy). Apart from Wilson disease, there are other treatable metabolic diseases that typically present with dystonia or ataxia but in which patients are occasionally reported to have tremor. These include Niemann-Pick type C, glutaric aciduria type 1, ataxia with vitamin E deficiency, Segawa disease and coenzyme Q10 deficiency (table 1).^57–59^ In case of a tremor associated with neuropathy, test for acquired causes, including paraproteinemia.

### Step 6: is an MRI indicated?

A cerebral MRI scan is worthwhile when the differential diagnosis includes an aetiology resulting in structural abnormalities. As a guiding principle, we propose to perform an MRI in patients with a *combined* tremor syndrome that is 1) focal/unilateral, 2) non-classical in appearance, 3) in case there is a sudden onset or stepwise deterioration or 4) a family history of movement disorders combined with cognitive or psychiatric symptoms. The latter suggests a heritable neurodegenerative or metabolic disorder, which can sometimes be identified using MRI. Look for lesions or atrophy in the basal ganglia, cerebellum and brainstem. Based on the clinical characteristics identified in step 2, specific signs to look for include hummingbird sign, morning glory sign and hot cross bun sign in atypical parkinsonism^60^ (combined with bradykinesia); panda sign in Wilson disease^61^ (combined with dystonia) and middle cerebral peduncle and splenium sign in fragile X-associated tremor ataxia syndrome (FXTAS, combined with ataxia).^22 62^ Generally, patients with an *isolated* tremor syndrome will have normal MRI scans and thus structural imaging is not indicated. However, this rule of thumb should be abandoned in isolated patients with tremor in case of either hints at an acquired cause (sudden onset or stepwise deterioration, unilateral tremor), or a family history of movement disorders combined with cognitive or psychiatric symptoms. A brain MRI is unnecessary to diagnose a functional tremor, but we recognise that it is sometimes needed to reassure a patient who has remaining fears about an alternative diagnosis despite the neurologist’s explanation.^63^

### Step 7: is presynaptic dopamine transporter imaging indicated?

Presynaptic dopamine transporter imaging (123I-FP-CIT SPECT (DaT-SPECT)) can be helpful in some patients with tremor. Dopamine receptor imaging (such as F-DOPA PET) can also be used, but this is much less common in most clinics. Here, we propose guiding principles on indications for nuclear imaging: these are not extensively evidence-based due to a lack of good data and the absence of gold standard for many tremor syndromes. Because this technique is aimed at assessing the presence of a presynaptic dopaminergic deficit, it will only be relevant if we wish to differentiate tremor related to parkinsonism from other tremor syndromes, and considerable doubt remains after steps 1–6 have been taken. More specifically, presynaptic dopamine transporter imaging is well suited to distinguish between parkinsonism-associated tremor and ET,^64^ and dystonic tremor, which can be a cause of scans without evidence of dopaminergic deficit in suspected patients with PD.^65^ However, although presynaptic dopamine transporter imaging is used to distinguish drug-induced parkinsonism from neurodegenerative forms of parkinsonism,^66^ it cannot reliably make a distinction between the different causes of a parkinsonism-associated tremor.^67^

### Step 8: are genetic tests indicated?

Genetic testing can be considered but should be limited to particular situations. There are several genetic mutations that can cause a clinical syndrome that includes tremor. However, in a patient presenting with *isolated* tremor, there is currently no indication for genetic testing as no disease-causing genes are known for isolated tremor syndromes. This underlines the importance of clinical examination and particularly the differentiation in *isolated* versus *combined* tremor syndromes, as depicted in figure 2.

Genetic testing can be considered in patients with a *combined tremor* and a family history of movement disorders, cognitive symptoms, psychiatric symptoms or neuropathy. For instance, a familial syndrome consisting of tremor and ataxia could point towards FXTAS^68^ or a spinocerebellar atrophy (SCA).^69^ In Klinefelter syndrome, a predominantly postural and kinetic tremor has been described, sometimes with dystonic features.^70^ More rarely, a combination of tremor and ataxia can be found in patients with adult ataxia telangiectasia^71^ or inborn errors of metabolism such as Niemann-Pick type C^72^ (figure 2). Tremor can also be a feature in the clinical presentation of some of the early onset/familial parkinsonisms.^73^ In familial cases of tremor and dystonia, DYT3 (Lubag’s disease)^74^ and DYT24 (ANO3)^75^ can be considered, as well as metabolic disorders (table 2). Particularly consider Wilson disease^55^ and Niemann-Pick type C if the movement disorders coincide with cognitive or psychiatric symptoms. Moreover, in patients with familial neuropathy and tremor, several Charcot-Marie-Tooth variants are possible,^19^ as well as spinal bulbar muscle atrophy (Kennedy’s disease).^76^ Overall, these examples illustrate that genetic testing is only feasible in patients with a combined tremor syndrome and a relevant family history.

**Table 2:** Treatable metabolic disorders that may evoke tremor^57–59^

Metabolic disorder	Movement disorders	Laboratory tests	Gene
**Wilson disease**	Dystonia, parkinsonism, ataxia, chorea, tremor	Serum ceruloplasmin, 24 hours urinary copper excretion	ATP7B
**Niemann-Pick type C**	Ataxia, dystonia, *rarely tremor*	Oxysterols, chitotriosidase	NPC1, NPC2
**Glutaric aciduria type 1**	Dystonia, parkinsonism, chorea, *rarely tremor*	Plasma+urine organic acids, plasma acylcarnitines	GCDH
**Ataxia with vitamin E deficiency**	Ataxia, dystonia, *rarely tremor*	Plasma vitamin E level	TTPA
**Segawa disease (DRD)**	Dystonia, parkinsonism, tremor	CSF dopamine levels	GCH1
**Coenzyme Q10 deficiency**	Ataxia, dystonia, tremor, spasticity	Serum lactate, biochemical activities of respiratory chain complexes in skin or muscle, muscle CoQ10 level	Multiple genes

CSF, cerebrospinal fluid; DRD, dopa-responsive dystonia.

In some instances, a particular genetic diagnosis will be suspected and tested by means of conventional Sanger sequencing, but if the list of possible genes is long next-generation sequencing will be the method of choice. This can be done either by using whole-genome sequencing (WGS), whole exome sequencing (WES) or targeted resequencing (TRS) panels that focus on a selection of known disease-causing genes. An advantage of diagnostic gene panels over WGS and WES is that it evades the dilemmas connected to the discovery of unsolicited findings. However, a targeted gene panel needs to be continuously updated with regard to newly established disease genes to be equally effective as WGS or WES, and a tremor gene panel may not be in place. Moreover, be aware that next-generation sequencing is not sensitive for repeat expansions (such as FXTAS, SCAs) and mitochondrial DNA mutations (such as sensory ataxia with neuropathy, dysarthria and ophthalmoparesis). Currently, the cost of next-generation sequencing techniques is falling rapidly and comparable to that of sequencing three individual genes. For further guidance, we advise soliciting a clinical geneticist for patients in whom genetic testing is indicated.

## Conclusions

We have proposed a stepwise diagnostic approach to patients presenting with upper limb tremor, to guide clinicians in establishing a clinical tremor syndrome and performing the relevant tests to determine its aetiology. The proposed diagnostic algorithm is in line with the latest Consensus Statement^2^ and encourages clinicians to make a distinction between isolated and combined tremor syndromes, based on clinical features. This is key, as this distinction subsequently informs our choices for diagnostic testing. We believe that our diagnostic algorithm will be useful to practising neurologists whether they have expertise in the field of movement disorders or not. The stepwise setup tapers diagnostic tests, starting with those tests that are easily available and indicated in most patients, and finishing with more specialised diagnostic procedures, including considerations for next-generation genetic testing. By following the presented steps, the long list of possible causes for tremor is reduced to manageable portions. We hope that the approach proposed in this article will decrease diagnostic uncertainty and increase the diagnostic yield in patients with tremor.

## References

[1] Louis ED, Ferreira JJ How common is the most common adult movement disorder? update on the worldwide prevalence of essential tremor. Mov Disord 2010;25:534–41. 10.1002/mds.22838 20175185

[2] Bhatia KP, Bain P, Bajaj N, et al Consensus statement on the classification of tremors. from the task force on tremor of the International Parkinson and movement disorder Society. Mov Disord 2018;33:75–87. 10.1002/mds.27121 29193359 PMC6530552

[3] Nieuwhof F, Panyakaew P, van de Warrenburg BP, et al The patchy tremor landscape: recent advances in pathophysiology. Curr Opin Neurol 2018;31:455–61. 10.1097/WCO.0000000000000582 29750732

[4] Deuschl G, Bain P, Brin M Consensus statement of the movement disorder Society on tremor. AD hoc scientific Committee. Mov Disord 1998;13 Suppl 3:2–23. 10.1002/mds.870131303 9827589

[5] Zutt R, van Egmond ME, Elting JW, et al A novel diagnostic approach to patients with myoclonus. Nat Rev Neurol 2015;11:687–97. 10.1038/nrneurol.2015.198 26553594

[6] Shaikh AG, Jinnah HA, Tripp RM, et al Irregularity distinguishes limb tremor in cervical dystonia from essential tremor. J Neurol Neurosurg Psychiatry 2008;79:187–9. 10.1136/jnnp.2007.131110 17872981 PMC2737356

[7] Schwingenschuh P, Deuschl G Functional tremor. Handb Clin Neurol 2016;139:229–33. 10.1016/B978-0-12-801772-2.00019-9 27719841

[8] Pareés I, Kojovic M, Pires C, et al Physical precipitating factors in functional movement disorders. J Neurol Sci 2014;338:174–7. 10.1016/j.jns.2013.12.046 24439198

[9] Raethjen J, Kopper F, Govindan RB, et al Two different pathogenetic mechanisms in psychogenic tremor. Neurology 2004;63:812–5. 10.1212/01.wnl.0000137012.35029.6b 15365128

[10] Dirkx MF, Zach H, Bloem BR, et al The nature of postural tremor in Parkinson disease. Neurology 2018;90:e1095–103. 10.1212/WNL.0000000000005215 29476038 PMC5880634

[11] Zach H, Dirkx M, Bloem BR, et al The clinical evaluation of Parkinson's tremor. J Parkinsons Dis 2015;5:471–4. 10.3233/JPD-150650 26406126 PMC4923747

[12] Jankovic J, Schwartz KS, Ondo W Re-Emergent tremor of Parkinson ’ S disease, 1999: 646–50. 10.1136/jnnp.67.5.646 PMC1736624 10519872

[13] Tison F, Yekhlef F, Chrysostome V, et al Parkinsonism in multiple system atrophy: natural history, severity (UPDRS-III), and disability assessment compared with Parkinson's disease. Mov Disord 2002;17:701–9. 10.1002/mds.10171 12210859

[14] Fujioka S, Algom AA, Murray ME, et al Tremor in progressive supranuclear palsy. Parkinsonism Relat Disord 2016;27:93–7. 10.1016/j.parkreldis.2016.03.015 27039056 PMC4887294

[15] Defazio G, Gigante AF, Abbruzzese G, et al Tremor in primary adult-onset dystonia: prevalence and associated clinical features. J Neurol Neurosurg Psychiatry 2013;84:404–8. 10.1136/jnnp-2012-303782 23142961

[16] Erro R, Rubio-Agusti I, Saifee TA, et al Rest and other types of tremor in adult-onset primary dystonia. J Neurol Neurosurg Psychiatry 2014;85:965–8. 10.1136/jnnp-2013-305876 24249781 PMC4145451

[17] Defazio G, Conte A, Gigante AF, et al Is tremor in dystonia a phenotypic feature of dystonia? Neurology 2015;84:1053–9. 10.1212/WNL.0000000000001341 25663232

[18] Masuhr F, Wissel J, Müller J, et al Quantification of sensory trick impact on tremor amplitude and frequency in 60 patients with head tremor. Mov Disord 2000;15:960–4. 10.1002/1531-8257(200009)15:5&lt;960::aid-mds1029&gt;3.0.co;2-g 11009205

[19] Saifee TA, Schwingenschuh P, Reilly MM, et al Tremor in inflammatory neuropathies. J Neurol Neurosurg Psychiatry 2013;84:1282–7. 10.1136/jnnp-2012-303013 22952325

[20] Hashimoto T, Sato H, Shindo M, et al Peripheral mechanisms in tremor after traumatic neck injury. J Neurol Neurosurg Psychiatry 2002;73:585–7. 10.1136/jnnp.73.5.585 12397157 PMC1738132

[21] Stavusis J, Lace B, Schäfer J, et al Novel mutations in MyBPC1 are associated with myogenic tremor and mild myopathy. Ann Neurol 2019;86:129-142. 10.1002/ana.25494 31025394 PMC6685440

[22] Ure RJ, Dhanju S, Lang AE, et al Unusual tremor syndromes: know in order to recognise. J Neurol Neurosurg Psychiatry 2016;87:1191–203. 10.1136/jnnp-2015-311693 26985048

[23] Baizabal-Carvallo JF, Cardoso F, Jankovic J Myorhythmia: phenomenology, etiology, and treatment. Mov Disord 2015;30:171–9. 10.1002/mds.26093 25487777

[24] Jankovic J, Tintner R Dystonia and parkinsonism. Parkinsonism Relat Disord 2001;8:109–21. 10.1016/S1353-8020(01)00025-6 11489676

[25] Paviolo JP et al Holmes tremor. Neurology 2016;86:931–8. 26865524 10.1212/WNL.0000000000002440 PMC4782118

[26] Hopfner F, Erhart T, Knudsen K, et al Testing for alcohol sensitivity of tremor amplitude in a large cohort with essential tremor. Parkinsonism Relat Disord 2015;21:848–51. 10.1016/j.parkreldis.2015.05.005 26002382

[27] Haubenberger D, Hallett M Essential tremor. N Engl J Med 2018;378:1802–10. 10.1056/NEJMcp1707928 29742376

[28] van der Stouwe AMM, Elting JW, van der Hoeven JH, et al How typical are 'typical' tremor characteristics? Sensitivity and specificity of five tremor phenomena. Parkinsonism Relat Disord 2016;30:23–8. 10.1016/j.parkreldis.2016.06.008 27346607

[29] Cohen O, Pullman S, Jurewicz E, et al Rest tremor in patients with essential tremor. Arch Neurol 2003;60:405 10.1001/archneur.60.3.405 12633153

[30] Stolze H, Petersen G, Raethjen J, et al The gait disorder of advanced essential tremor. Brain 2001;124:2278–86. 10.1093/brain/124.11.2278 11673328

[31] Rajalingam R, Breen DP, Lang AE, et al Essential tremor plus is more common than essential tremor: insights from the reclassification of a cohort of patients with lower limb tremor. Parkinsonism Relat Disord 2018;56:109–10. 10.1016/j.parkreldis.2018.06.029 29958776

[32] Fearon C, Espay AJ, Lang AE, et al Soft signs in movement disorders: friends or foes? J Neurol Neurosurg Psychiatry 2019;90:961–2. 10.1136/jnnp-2018-318455 30409889

[33] Rajan R, Pandey S, Anandapadmanabhan R, et al Interrater and intrarater agreement on the 2018 consensus statement on classification of tremors. Mov Disord 2018;33:1966-1967. 10.1002/mds.27513 30329183

[34] Gövert F, Becktepe J, Balint B, et al Temporal discrimination is altered in patients with isolated asymmetric and jerky upper limb tremor. Mov Disord 2020;35:306-315. 10.1002/mds.27880 31724777

[35] Louis ED, Bares M, Benito-Leon J, et al Essential tremor-plus: a controversial new concept. Lancet Neurol 2020;19:266-270. 10.1016/S1474-4422(19)30398-9 31767343 PMC10686582

[36] Vidailhet M Essential tremor-plus: a temporary label. Lancet Neurol 2020;19:202-203. 10.1016/S1474-4422(19)30442-9 31767342

[37] Schnitzler A, Gross J Normal and pathological oscillatory communication in the brain. Nat Rev Neurosci 2005;6:285–96. 10.1038/nrn1650 15803160

[38] Elble RJ Characteristics of physiologic tremor in young and elderly adults. Clin Neurophysiol 2003;114:624–35. 10.1016/s1388-2457(03)00006-3 12686271

[39] Shibasaki H, Yamashita Y, Kuroiwa Y Electroencephalographic studies myoclonus. Brain 1978;101:447–60. 10.1093/brain/101.3.447 101279

[40] van Rootselaar A-F, van Schaik IN, van den Maagdenberg AMJM, et al Familial cortical myoclonic tremor with epilepsy: a single syndromic classification for a group of pedigrees bearing common features. Mov Disord 2005;20:665–73. 10.1002/mds.20413 15747356

[41] Zutt R, Elting JW, van Zijl JC, et al Electrophysiologic testing AIDS diagnosis and subtyping of myoclonus. Neurology 2018;90:e647–57. 10.1212/WNL.0000000000004996 29352095 PMC5818165

[42] Mameniškienė R, Wolf P Epilepsia partialis continua: a review. Seizure 2017;44:74–80. 10.1016/j.seizure.2016.10.010 28029552

[43] Schwingenschuh P, Saifee TA, Katschnig-Winter P, et al Validation of "laboratory-supported" criteria for functional (psychogenic) tremor. Mov Disord 2016;31:555–62. 10.1002/mds.26525 26879346

[44] O'Suilleabhain PE, Matsumoto JY Time-frequency analysis of tremors. Brain 1998;121 (Pt 11:2127–34. 10.1093/brain/121.11.2127 9827772

[45] Mehanna R, Jankovic J Movement disorders in cerebrovascular disease. Lancet Neurol 2013;12:597–608. 10.1016/S1474-4422(13)70057-7 23602779

[46] Vial F, Kassavetis P, Merchant S, et al How to do an electrophysiological study of tremor. clinical neurophysiology practice. Clinical Neurophysiology Practice;4:134–42. 31886436 10.1016/j.cnp.2019.06.002 PMC6923291

[47] Deuschl G, Raethjen J, Lindemann M, et al The pathophysiology of tremor. Muscle Nerve 2001;24:716–35. 10.1002/mus.1063 11360255

[48] van der Stouwe AMM, Elting JW, van der Hoeven JH, et al How typical are ‘typical’ tremor characteristics? Sensitivity and specificity of five tremor phenomena. Parkinsonism Relat Disord 2016;30:23–8. 10.1016/j.parkreldis.2016.06.008 27346607

[49] Longardner K, Undurraga FV, Nahab FB, et al How do I assess tremor using novel technology? Mov Disord Clin Pract 2019;6:733–4. 10.1002/mdc3.12818 31745492 PMC6856460

[50] Koch M, Mostert J, Heersema D, et al Tremor in multiple sclerosis. J Neurol 2007;254:133–45. 10.1007/s00415-006-0296-7 17318714 PMC1915650

[51] Gajos A, Bogucki A, Schinwelski M, et al The clinical and neuroimaging studies in Holmes tremor. Acta Neurol Scand 2010;122:360–6. 10.1111/j.1600-0404.2009.01319.x 20078445

[52] Morgan JC, Sethi KD Drug-Induced tremors. Lancet Neurol 2005;4:866–76. 10.1016/S1474-4422(05)70250-7 16297844

[53] Lucchini RG, Hashim D Tremor secondary to neurotoxic exposure: mercury, lead, solvents, pesticides. Handb Clin Neurol 2015;131:241–9. 10.1016/B978-0-444-62627-1.00014-7 26563793

[54] Lance JW, Hinterberger H Symptoms of pheochromocytoma, with particular reference to headache, correlated with catecholamine production. Arch Neurol 1976;33:281–8. 10.1001/archneur.1976.00500040065011 1259642

[55] Roberts EA, Schilsky ML, American Association for Study of Liver Diseases (AASLD) Diagnosis and treatment of Wilson disease: an update. Hepatology 2008;47:2089–111. 10.1002/hep.22261 18506894

[56] Kruer MC, Boddaert N Neurodegeneration with brain iron accumulation: a diagnostic algorithm. Semin Pediatr Neurol 2012;19:67–74. 10.1016/j.spen.2012.04.001 22704259 PMC3381651

[57] Ebrahimi-Fakhari D, Van Karnebeek C, Münchau A Movement disorders in treatable inborn errors of metabolism. Mov Disord 2019;34:598–613. 10.1002/mds.27568 30557456

[58] Jinnah HA, Albanese A, Bhatia KP, et al Treatable inherited rare movement disorders. Mov Disord 2018;33:21–35. 10.1002/mds.27140 28861905 PMC5921079

[59] Emmanuele V, López LC, Berardo A, et al Heterogeneity of Coenzyme Q _10_ Deficiency. Arch Neurol 2012;69:978–83. 10.1001/archneurol.2012.206 22490322 PMC3639472

[60] Meijer FJA, Goraj B, Bloem BR, et al Clinical application of brain MRI in the diagnostic work-up of parkinsonism. J Parkinsons Dis 2017;7:211–7. 10.3233/JPD-150733 28282809 PMC5438480

[61] Prashanth LK, Sinha S, Taly AB, et al Do MRI features distinguish Wilson's disease from other early onset extrapyramidal disorders? an analysis of 100 cases. Mov Disord 2010;25:672–8. 10.1002/mds.22689 20437536

[62] Hall DA, Robertson E, Shelton AL, et al Update on the clinical, radiographic, and neurobehavioral manifestations in FXTAS and FMR1 premutation carriers. The Cerebellum 2016;15:578–86. 10.1007/s12311-016-0799-4 27287737 PMC7608648

[63] Stone J, Edwards M Trick or treat? showing patients with functional (psychogenic) motor symptoms their physical signs. Neurology 2012;79:282–4. 10.1212/WNL.0b013e31825fdf63 22764261

[64] Benamer HTS, Patterson J, Grosset DG, et al Accurate differentiation of parkinsonism and essential tremor using visual assessment of [123I]-FP-CIT SPECT imaging: The [123I]-FP-CIT study group. Mov. Disord. 2000;15:503–10. 10.1002/1531-8257(200005)15:3<503::AID-MDS1013>3.0.CO;2-V 10830416

[65] Schwingenschuh P, Ruge D, Edwards MJ, et al Distinguishing SWEDDs patients with asymmetric resting tremor from Parkinson's disease: a clinical and electrophysiological study. Mov Disord 2010;25:560–9. 10.1002/mds.23019 20131394 PMC2996567

[66] Lorberboym M, Treves TA, Melamed E, et al [123I]-FP/CIT SPECT imaging for distinguishing drug-induced parkinsonism from Parkinson's disease. Mov Disord 2006;21:510–4. 10.1002/mds.20748 16250023

[67] Kägi G, Bhatia KP, Tolosa E The role of DAT-SPECT in movement disorders. J Neurol Neurosurg Psychiatry 2010;81:5–12. 10.1136/jnnp.2008.157370 20019219

[68] Hall DA, Robertson E, Shelton AL, et al Update on the clinical, radiographic, and neurobehavioral manifestations in FXTAS and FMR1 premutation carriers. Cerebellum 2016;15:578–86. 10.1007/s12311-016-0799-4 27287737 PMC7608648

[69] Gan S-R, Wang J, Figueroa KP, et al Postural tremor and ataxia progression in spinocerebellar ataxias. Tremor Other Hyperkinet Mov 2017;7:492. 10.7916/D8GM8KRH PMC5647398 29057148

[70] Rabin ML, Mittal SO, Jabbari B Tremor and Klinefelter's syndrome. Tremor Other Hyperkinet Mov 2015;5:304. 10.7916/D84M93KR PMC4473154 26175955

[71] Méneret A, Ahmar-Beaugendre Y, Rieunier G, et al The pleiotropic movement disorders phenotype of adult ataxia-telangiectasia. Neurology 2014;83:1087–95. 10.1212/WNL.0000000000000794 25122203

[72] Anheim M, Lagha-Boukbiza O, Fleury-Lesaunier M-C, et al Heterogeneity and frequency of movement disorders in juvenile and adult-onset Niemann-Pick C disease. J Neurol 2014;261:174–9. 10.1007/s00415-013-7159-9 24178705

[73] Ferreira M, Massano J An updated review of Parkinson's disease genetics and clinicopathological correlations. Acta Neurol Scand 2017;135:273–84. 10.1111/ane.12616 27273099

[74] Evidente VGH, Advincula J, Esteban R, et al Phenomenology of "Lubag" or X-linked dystonia-parkinsonism. Mov Disord 2002;17:1271–7. 10.1002/mds.10271 12465067

[75] Stamelou M, Charlesworth G, Cordivari C, et al The phenotypic spectrum of DYT24 due to ANO3 mutations. Mov Disord 2014;29:928–34. 10.1002/mds.25802 24442708 PMC4150528

[76] Finsterer J Bulbar and spinal muscular atrophy (Kennedy's disease): a review. Eur J Neurol 2009;16:556–61. 10.1111/j.1468-1331.2009.02591.x 19405197

